# Partial Distal Ureteral Obstruction After Pediatric Kidney Transplantation: A Diagnostic Challenge

**DOI:** 10.7759/cureus.106471

**Published:** 2026-04-05

**Authors:** Sebastian G Tobia González, Jimena Krikorian, Anabella Maiolo

**Affiliations:** 1 Urology, Sor Maria Ludovica Children's Hospital, La Plata, ARG; 2 Urology, Driscoll Children's Hospital, Corpus Christi, USA; 3 Pediatric Surgery, Texas A&M College of Medicine, Corpus Christi, USA

**Keywords:** distal ureter, graft dysfunction, hydronephrosis, kidney transplantation, pediatric transplantation, ureteral ischemia, ureteral obstruction, ureteral reimplantation, ureteral stenosis

## Abstract

Ureteral complications are a significant source of morbidity after kidney transplantation. While complete ureteral obstruction is typically identified with standard imaging, partial obstruction may be more difficult to diagnose. We report the case of an eight-year-old girl who developed progressive hydronephrosis and graft dysfunction following kidney transplantation. Imaging studies, including ultrasound and retrograde pyelography, were inconclusive and failed to clearly localize the obstruction. Due to persistent graft dysfunction, surgical exploration was performed, revealing a short-segment distal ureteral stenosis approximately 1 cm from the vesical implantation site, consistent with segmental ischemia. The ureterovesical anastomosis was intact. Ureteral reimplantation resulted in the resolution of obstruction and recovery of graft function. Partial ureteral obstruction may be difficult to detect with standard imaging and should be considered in cases of unexplained graft dysfunction.

## Introduction

Ureteral complications following kidney transplantation are a well-recognized source of morbidity, with reported rates of ureteral stenosis ranging from 2% to 10% [1,2]. These complications most commonly present as complete obstruction and are typically identified using standard imaging modalities [3]. However, partial ureteral obstruction represents a less commonly described entity and may pose a diagnostic challenge.

Delayed diagnosis of ureteral obstruction can lead to progressive graft dysfunction and potentially irreversible damage [1]. Therefore, early recognition and appropriate management are critical [2]. We present a case of partial distal ureteral obstruction following pediatric kidney transplantation, highlighting the diagnostic limitations of conventional imaging and the importance of clinical suspicion [3].

## Case presentation

An eight-year-old girl underwent kidney transplantation for end-stage renal disease secondary to hemolytic uremic syndrome. The ureter was implanted using the Taguchi technique over a 4.7 Fr double-J stent, which was removed 30 days postoperatively. At that time, serum creatinine was 0.8 mg/dL.

At two months post-transplant, the patient developed hydronephrosis (renal pelvis 2.1 cm) associated with a rise in serum creatinine to 1.8 mg/dL, with partial improvement after hydration. Hydronephrosis persisted on follow-up imaging.

**Figure 1:**
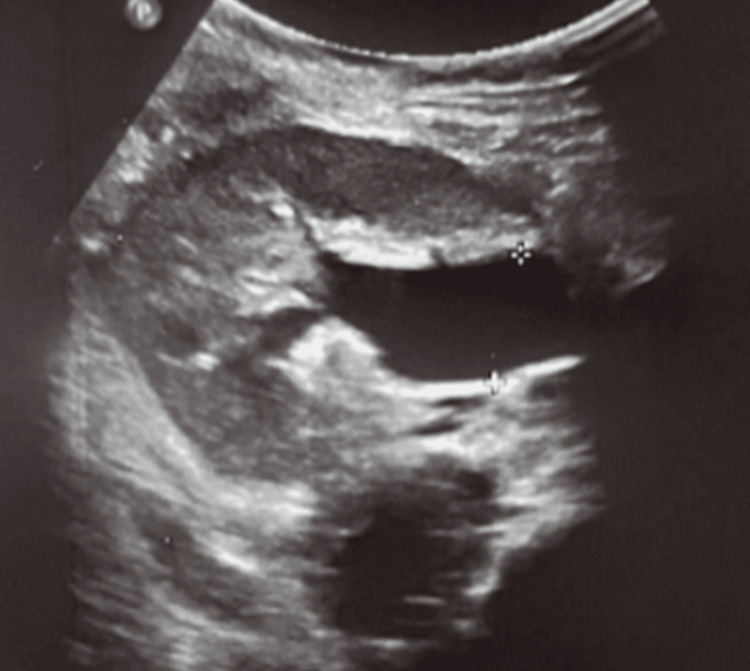
Ultrasound at four months post-transplant demonstrating persistent hydronephrosis (1.9 cm) without clear ureteral dilation

At four months, ultrasound demonstrated stable hydronephrosis (1.9 cm) without clear evidence of ureteral dilation. At six months post-transplant, serum creatinine increased again, prompting further evaluation (Figure 1). Renal biopsy excluded rejection.

Repeat imaging demonstrated distal ureteral dilation (0.8 cm) up to the bladder wall, raising suspicion for distal ureteral obstruction.

Endoscopic evaluation revealed a normal bladder and graft ureteral orifice. Retrograde pyelography demonstrated only a short segment of distal ureter (approximately 1.5 cm) without contrast passage proximally, suggesting obstruction but failing to define its nature.

**Figure 2:**
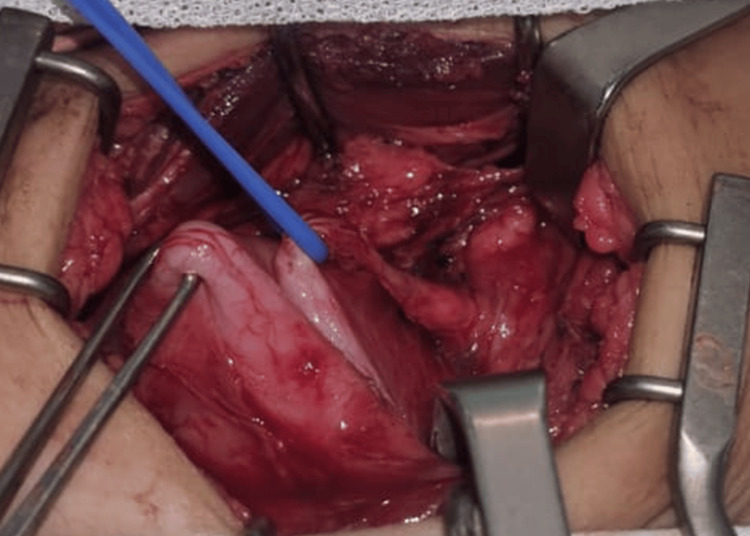
Intraoperative image showing distal ureteral stenosis near the vesical implantation site

Due to persistent graft dysfunction and inconclusive imaging findings, surgical exploration was performed. A short-segment partial distal ureteral stenosis, located approximately 1 cm from the vesical implantation site and extending over approximately 3 cm, was identified (Figure 2). The ureterovesical anastomosis appeared intact, indicating that the obstruction was non-anastomotic and most consistent with a segmental distal ureteral ischemic process.

**Figure 3:**
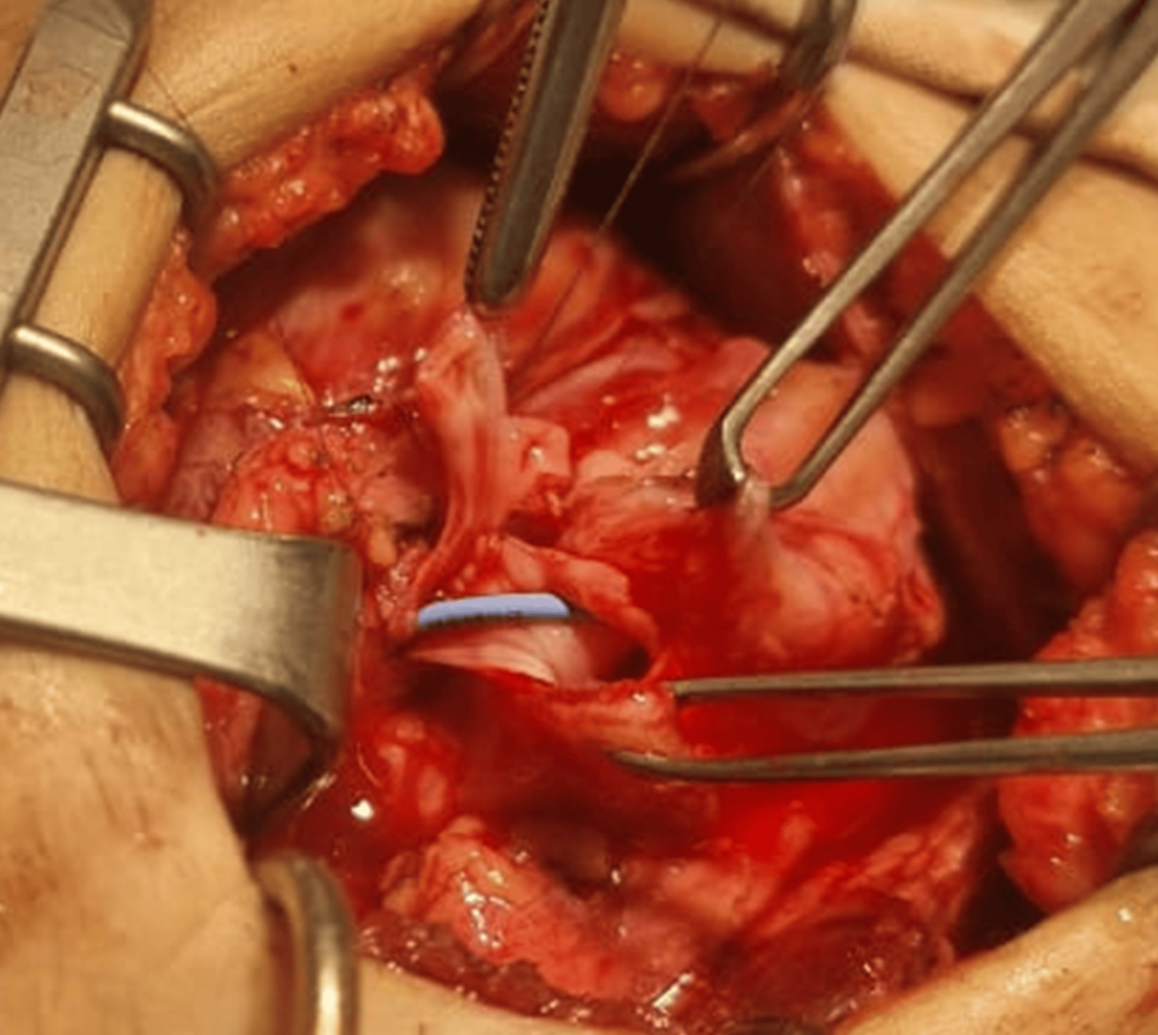
Reconstructed ureter with double-J stent in place following ureteral reimplantation

The distal ureter was partially opened, confirming patency toward the bladder and obstruction toward the graft. The stenotic segment was resected, and ureteral reimplantation was performed using the Lich-Gregoir technique over a double-J stent (Figure 3).

Postoperatively, hydronephrosis resolved and renal function normalized. Following surgical intervention, the patient demonstrated progressive clinical improvement with the resolution of hydronephrosis on follow-up imaging. Renal function remained stable, and no further obstructive complications were observed. At the six-month follow-up, the patient remained asymptomatic with preserved graft function.

## Discussion

Ureteral complications remain among the most frequent urological issues following kidney transplantation, with reported incidences ranging between 2% and 10% depending on surgical technique, follow-up duration, and patient population [1]. These complications include ureteral stenosis, urinary leakage, and obstruction, all of which may compromise graft function if not promptly recognized and treated [2]. In pediatric populations, additional anatomical and technical considerations may further influence outcomes, emphasizing the importance of early detection and tailored management strategies [3].

Ureteral stenosis, as observed in our case, is typically attributed to ischemia of the distal ureter, excessive ureteral dissection, or technical factors related to ureteroneocystostomy [3-5]. The distal ureter is particularly vulnerable due to its tenuous blood supply, making the preservation of periureteral tissue critical during transplantation [4]. Prior studies have consistently identified surgical technique, donor type, and ureteral handling as key determinants of postoperative ureteral complications [6,7].

The temporal evolution in this case, from isolated hydronephrosis to progressive distal ureteral dilation, highlights the value of serial imaging in the postoperative surveillance of transplant recipients. Ultrasound remains the first-line modality for detecting hydronephrosis and guiding further evaluation, given its non-invasive nature and accessibility [3]. Early identification of progressive dilation, particularly when associated with distal ureteral changes, should raise suspicion for obstructive pathology such as ureteral stenosis.

Management of ureteral complications after kidney transplantation depends on the severity and timing of presentation. Initial conservative or minimally invasive approaches, including stent placement or percutaneous interventions, may be appropriate in selected cases [8]. However, definitive surgical management is often required in cases of persistent or severe obstruction [9]. Ureteral reimplantation remains the gold standard for distal ureteral stenosis, offering high success rates and durable outcomes [10].

The use of ureteral stents at the time of transplantation has been widely studied as a preventive strategy. Evidence from randomized trials and systematic reviews suggests that routine stenting may reduce the incidence of early urological complications, although it may also increase the risk of urinary tract infections [11]. In our case, the placement of a double-J stent following ureteral reconstruction likely contributed to maintaining patency and facilitating the healing of the anastomosis.

This case underscores the importance of maintaining a high index of suspicion for ureteral complications in patients with persistent or progressive hydronephrosis after transplantation. While many cases of early hydronephrosis may be transient, the presence of distal ureteral dilation should prompt further evaluation to exclude obstruction. The integration of clinical findings, imaging progression, and timely surgical intervention is essential to preserve graft function and optimize patient outcomes.

Although ureteral complications are well-described in the literature, variations in presentation and timing continue to pose diagnostic challenges [2,12]. Our case contributes to the existing body of evidence by illustrating a clear radiologic progression culminating in surgically confirmed distal ureteral stenosis, reinforcing the need for structured follow-up protocols and early intervention when indicated.

## Conclusions

Partial distal ureteral obstruction following pediatric kidney transplantation represents a diagnostic challenge that may not be detected by standard imaging modalities. Segmental distal ureteral ischemia, even in the absence of anastomotic involvement, should be considered in cases of unexplained graft dysfunction, particularly when imaging findings are inconclusive and clinical suspicion remains high.
